# Predicting the risk of atherosclerotic cardiovascular disease among adults living with HIV/AIDS in Addis Ababa, Ethiopia: A hospital-based study

**DOI:** 10.1371/journal.pone.0260109

**Published:** 2021-11-29

**Authors:** Minyahil Woldu, Omary Minzi, Workineh Shibeshi, Aster Shewaamare, Ephrem Engidawork

**Affiliations:** 1 Department of Clinical Pharmacy and Pharmacology, Muhimbili University of Health and Allied Sciences, Dar es Salaam, Tanzania; 2 Department of Pharmacology and Clinical Pharmacy, School of Pharmacy, College of Health Sciences, Addis Ababa University, Addis Ababa, Ethiopia; 3 Zewditu Memorial Hospital, ART Clinic, Addis Ababa, Ethiopia; Centro Cardiologico Monzino, ITALY

## Abstract

**Background:**

Atherosclerotic Cardiovascular Disease (ASCVD) is an emerging problem among People living with HIV/AIDS (PLWHA). The current study aimed at determining the risk of ASCVD among PLWHA using the Pooled Cohort Equation (PCE) and the Framingham Risk score (FRS).

**Methods:**

A hospital-based study was carried out from January 2019 to February 2020 in PLWHA. The prevalence of ASCVD risk was determined in individuals aged between 20 to 79 and 40 to 79 years using the FRS and PCE as appropriate. Chi-square, univariate and multivariate logistic regressions were employed for analysis.

**Results:**

The prevalence of high-risk ASCVD for subjects aged 20 and above using both tools was 11.5 %. For those aged 40 to 79 years, PCE yielded an increased risk (28%) than FRS (17.7%). Using both tools; advanced age, male gender, smoking, and increased systolic blood pressure were associated with an increased risk of ASCVD. Younger age (adjusted odds ratio, AOR) 0.20, 95%CI: 0.004, 0.091; P< 0.001), lower systolic blood pressure (AOR 0.221, 95%CI: 0.074, 0.605 P< 0.004), and lower total cholesterol (AOR 0.270, 95%CI: 0.073, 0.997; p<0.049) were found to be independent predictors of reduced risk of ASCVD. Likewise, younger age (40 to 64 years), female gender, and lower systolic blood pressure were significantly associated with lower risk of ASCVD among patients aged 40 to 79 years using both PCE and FRS.

**Conclusions:**

A considerable number of PLWHA have been identified to be at risk for ASCVD. ASCVD risk was significantly associated with advanced age, male gender, higher blood pressure, and smoking using both FRS and PCE. These factors should therefore be taken into account for designing management strategies.

## Introduction

The Human Immunodeficiency Virus (HIV) associated morbidity and mortality have declined significantly since the introduction of Antiretroviral Therapy (ART) [1], which extended the life expectancy of People Living with HIV/AIDS (PLWHA). However, Cardiovascular Diseases (CVDs) and related other non-communicable diseases remain an increased concern among PLWHA [2, 3]. On top of this, the etiology of Atherosclerotic Cardiovascular Disease (ASCVD) in PLWHA is also multi-factorial [4]. Several risk factors for CVDs in PLWHA including age, dyslipidemia, diabetes mellitus, hypertension, family history, sedentary life, cigarette smoking, and cocaine use have been reported [5, 6].

Heart attack, ASCVD, stroke, and other forms of CVDs have been reported to be nearly doubled in PLWHA compared to the general population, despite well-control of HIV-infections with combination ARTs (cARTs) [7–9]. This could be attributed to complexity of the management as well as the need for lifetime intervention [10]. It is therefore prudent to adequately determine the risk of such CVDs for proper monitoring as well as improving outcomes of cART [11].

ASCVD has become the major factor, limiting life expectancy, and causing death in participants age 45 years and above [12]. The intensity of efforts to prevent CVDs depends on the absolute risk of ASCVD, which can be calculated either using the Pooled Cohort Equation (PCE) or the Framingham Risk Score (FRS) [13, 14]. PCE and FRS have been considered as reliable and accurate benchmark for assessing cardiovascular risks in the general population [15]. Indeed, the 10 year PCE & FRS estimations are the most widely used tools for ASCVD risk evaluation [16, 17] and both share similar variables to determine the risk, even though few variations exist [3, 10, 18].

The PCE was designed to determine the risk among population age 40 to 79 years, whereas the FRS was designed to evaluate ASCVD risk among people age 20 to 79 years as well as age 40 to 79 years [19, 20].

The risk can be classified as low-risk (<10%), moderate risk (10–20%), and high-risk (>20%) using FRS [21], and as low risk (<7.5%) and elevated-risk (≥ 7.5%) using PCE [18]. The prevalence of ASCVD is reported to be in the range of 70–90% for the low-risk, 20–30% for the moderate risk, and 0–20% for the high-risk using FRS [22–25]. The overall prevalence of elevated ASCVD risk using either of the tools was approximately 25% [26].

Although CVD is an emerging and significant cause for morbidity and mortality in HIV-infected patients, the main guidelines of HIV therapy are still focusing on HIV and opportunistic infections, with little or no emphasis on non-communicable diseases [27]. Moreover, though the issue is well investigated in developed countries, there is paucity of such data in resource-constrained regions such as sub-Saharan Africa, particularly Ethiopia. Thus, the present study aimed at determining the risk and outcome of ASCVD among PLWHA using PCE and FRS.

## Methods

### Study setting

This was a hospital-based study conducted in PLWHA on follow-up care between January 2019 and February 2020 at Zewditu Memorial Hospital, Addis Ababa, Ethiopia. It was the first hospital that commenced and initiated the subsidized fee-based scheme of ART service in Ethiopia in 2003. Currently, there are about 7,674 active adults and 2,558 children in its follow-up nest [28].

### Patients and sampling

Patients were sampled from newly registered as well as existing PLWHA on follow-up care at ZMH. Adult (aged 20 years and above) HIV positive patients and willing to participate were included. Severely ill patients as well as pregnant and breastfeeding women were excluded from the study. Sample size was calculated using the formula for descriptive studies: Sample size (n) = [DEFF*Np (1-p)] / [(d2/z21- α/2*(N-1) +p*(1-p)]: where N is population size (7674); P (≅ 25% ± 5%) is the estimated prevalence for ASCVD in HIV- infected population obtained from the literature [23]; DEEF, design effect (1.5); d, precision (0.1); and z21- α/2, 1.96. Considering 10% contingency (lost to follow-up and defaulters). An estimated sample size of 314 was obtained using the aforementioned assumptions. A systematic random sampling technique was used to recruit study participants. The sample interval (K) was calculated using the formula N/n (7674/314≅24). The first participant was selected using a lottery method from patients having an appointment during the first day. Since the number of adult ART clinics in the hospital were four; every six (24/4) volunteer participants were then enrolled in the study. Negative response and refusal to continue participation following prior consent was managed by enrolling the next participant automatically.

### Data collection

Detailed information about the participants was obtained through laboratory tests, clinical examination/measurement, patient interviews, and chart review. The instrument for a face-to-face interview was adapted from the structured questionnaire used by the WHO stepwise approach to non-communicable disease risk factor surveillance (STEPS-2014) [29]. The questionnaire included information related to socio-demographic characteristics (age, gender, religion, civil status, address, educational level, occupation, monthly income), clinical characteristics (Family History of CVD, Viral Load, CD4 Count, Time Since ART Initiated, WHO Staging, ART Medication Regimen, and Frequency of ART Medication Switch), tobacco use (active, passive and smoking history), alcohol consumption (active, alcohol use history), coffee, and khat use. The questionnaire was pre-tested prior to the actual data collection and appropriate modifications were performed accordingly.

The online version of PCE calculator (originally created by the American College of Cardiology/American Heart Association) was used with prior permission, and the online free version of the FRS tool (originally created by the National, Heart, Lung, and Blood Institute) was accessed from the GlobalRPH.com.

### Data analysis

Data were cleaned, coded, double-entered, and analyzed using IBM SPSS Statistics software version 25 for Windows. All categorical variables were coded as 0 (for female and no responses) and (1 for male and yes responses). To make comparison easier, the risk calculated using FRS was treated as dichotomous (low and elevated risk) by considering moderate and high risk as elevated risk. Hence, low-risk (<10% for FRS or <7.5% for PCE) was coded as “0” and elevated-risk (≥ 10% for FRS or ≥ 7.5% for PCE) as “1”. Permission was obtained from ClinCalc.com to use the online calculator.

Variables including age, gender, lipid profile (total cholesterol & HDL), systolic blood pressure, smoking status, and blood pressure medications were used to calculate the risk of ASCVD in both PCE and FRS. In addition, race for PCE and LDL for FRS were used [18, 30].

Determination of the risk of ASCVD among patients aged 20 to 79 years (n = 288) was carried out using the FRS tool. A separate determination of the risk for patients aged 40 to 79 years was also carried out using both tools.

Chi-square, univariate, bivariate, and multivariate log-linear regression analyses as well as the Pearson’s chi-square with continuity correction and odds ratio (OR) with the corresponding 95% confidence interval (CI) were used to estimate the relations, associations, and interactions between variables. The outputs of bivariate analysis with p-value ≤ 0.20 were further analyzed in multivariate logistic regression to control the effect of confounders. Statistical significance was considered at p-value ≤0.05.

### Ethics statement

The study was approved by several Institutional Review Boards, including the Muhimbili University of Health and Allied Sciences, Office of the Director of Research and Publications, Dar es Salaam, Tanzania (Ref. No. 2018-04-23/AEC//Vol. XII/88), School of Pharmacy (ERB/SOP/41/11/2018), College of Health Sciences (Meeting number 08/2018), Addis Ababa University, and City Government of Addis Ababa Health Bureau, (Ref no. A/A/HB/344438/227), Addis Ababa, Ethiopia. The study was carried out under the tenets of the Declaration of Helsinki. Patients provided written informed consent before they participated in the study. Confidentiality and anonymity were maintained by restricting data access and removing identifiers.

## Results

### Enrolment

**Fig 1:**
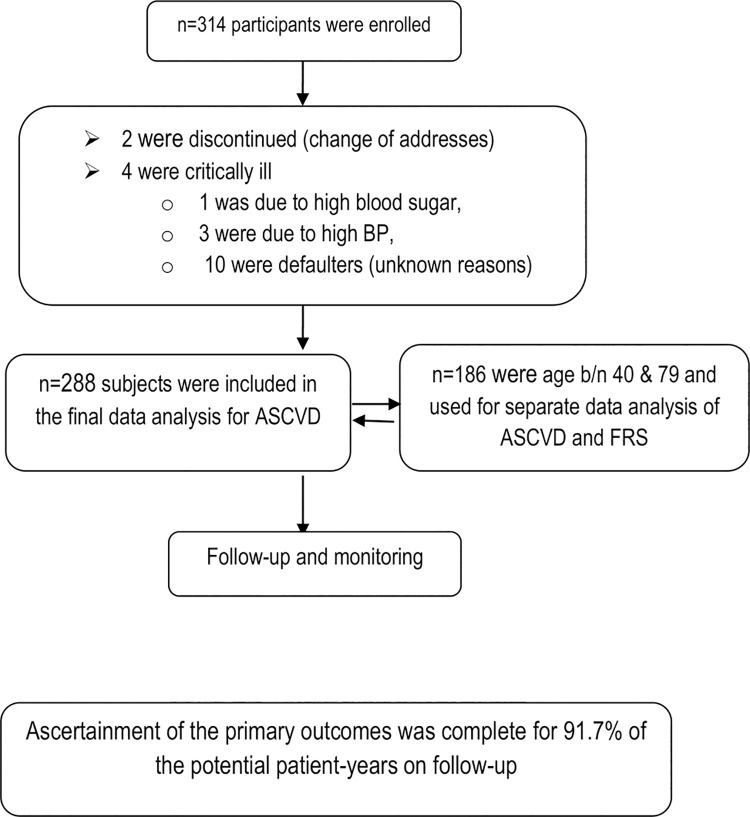
Enrolment, screening and follow up.

Initially, 314 patients were enrolled. However, 26 participants were later excluded for a variety of reasons: 10 were defaulter (unknown reasons); 4 due to critical illness (three due to high blood pressure, one due to high blood sugar); and 2 discontinued due to change of address (Fig 1). Hence, data for 288 patients were used for analysis.

### Sociodemographic and clinical characteristics

The sociodemographic and clinical characteristics of patients, as determined by FRS, are depicted in Table 1. Majority of the patients were female (69.4%) and under 50 years of age (69.4%). Close to half (45.1%) were married and a third of them (32.3%) completed high school education. About 12% of the patients had an elevated-risk for ASCVD. Chi-square analysis revealed that the male gender (93.9%) had a significantly elevated-risk (χ^2^ = 35.881, *p*<0.001) than the female gender (6.1%). Likewise, the risk increased with age, with patients aged ≥50 years (93.9%) having a significant elevated-risk for ASCVD (χ^2^ = 67.233, *p*<0.001) than those under 50 years of age. Although married participants (51.5%) and low-income generating group (81.8%) tended to have an increased risk for ASCVD, it failed to reach a statistical significance (Table 1).

**Table 1:** Socio-demographic characteristics of HIV-infected patients with 20 and above years of age on ART follow up care at Zewditu Memorial Hospital, Addis Ababa, Ethiopia.

Socio-demographic	ASCVD risk using FRS		χ^2^-value	P value
	Low risk (%)	Elevated risk		
**Number (%)**	255 (88.5)	33 (11.5)		
**Gender**				
Female	160 (62.7)	2 (6.1)	35.881*^a^	*p*<0.001
Male	95 (37.3)	31 (93.9)		
**Age**				
20–49	198 (77.6)	2 (6.1)	67.233*^a^	*p*<0.001
≥50	57 (22.4)	31 (93.9)		
**Civil status**				
Never married	49 (19.2)	4 (12.1)	1.138^#^^a^	*p* = 0.768
Married	113 (44.3)	17 (51.5)		
Divorced	55 (21.6)	7 (21.2)		
Widowed/r	38 (14.9)	5 (15.2)		
**Educational status**				
No formal education	34 (13.3)	1 (3.0)	7.944^#^^a^	*p* = 0.159
Primary (1-6^th^ grade)	58 (22.7)	7 (21.2)		
Secondary Junior (7-8^th^ grade)	24 (9.4)	3 (9.1)		
High school (9-12^th^ grade)	87 (34.1)	9 (27.3)		
College/university diploma	37 (14.5)	10 (30.3)		
College/ University Degree/master or above	15 (5.9)	3 (9.1)		
**Occupational status** ^‡^				
A-list (intermediate to high income)	38 (14.9)	6 (18.2)	5.211^#^^a^	*p* = 0.74
B-list (small to intermediate income)	171 (67.1)	16 (48.5)		
C-list (non-income generating)	46 (18)	11 (33.3)		

n = 288; Data presented as % prevalence

*Continuity Correction is computed for a 2x2 table

#Pearson Chi-square; ASCVD, atherosclerotic cardiovascular disease; FRS, Framingham Risk Score

^‡^ Classification is based on ISEC (International Socio-Economic Classes) [31]. A-List (Higher-level professional; Higher level manager and entrepreneur; Lower-level professional; Lower-level manager; Clerical routine non-manual worker; Sales and service routine non-manual worker). B-List (Small self-employed with employee; Small self-employed without employer; Small self-employed in agriculture; Manual supervisor; Skilled manual worker; Semi- and unskilled manual worker. C-List (Agricultural laborers; Retired; Students; unemployed).

### Association studies

The calculated risk using both tools is presented in Table 2. Accordingly, individual aged 65 to 79 years had a significantly higher risk for ASCVD than their younger compatriots (40–64 years) in both PCE (χ2 = 20.758, p<0.001), and FRS (χ2 = 28.207, p<0.001) methods. Characteristics such as blood group, WHO staging, and family history did not have significant contribution to ASCVD risk. Smoking was also identified as a significant predictor of ASCVD in both tools, though one cell had a count of less than 5% (Table 2). Hence, Fisher’s Exact Test was used in place of Pearson Chi-square.

**Table 2:** The risk for atherosclerosis cardiovascular disease using the Pooled Cohort Equation and the Framingham Risk Score in HIV-infected patients aged 40 to 79 years at Zewditu Memorial Hospital, Addis Ababa, Ethiopia.

Characteristics		ASCVD risk using PCE		χ^2^-value	P value	ASCVD risk using FRS		χ^2^-value	P value
		Low risk	Elevated risk			Low risk	Elevated risk		
**Number (%)**		134 (72)	52 (28)			153 (82.3)	33 (17.7)		
**Age (Year)**	40–64	134 (100)	43 (82.7)	20.758*^a^	p<0.001	139 (90.8)	17(51.5)	28.207*^a^	*p*<0.001
	65–79	0 (0)	9 (17.3)			14 (9.2)	16 (48.5)		
**Gender**	Female	72 (53.7)	13 (25.0)	11.331*^a^	*p* = 0.001	83 (54.2)	2 (6.1)	23.496*^a^	*p* = 0.001
	Male	62 (46.3)	39 (75.0)			70 (45.8)	31 (93.9)		
**Civil status**	Never married	18 (13.4)	5 (9.6)	0.679^#^^a^	*p* = 0.787	19 (12.4)	4 (12.1)	0.207^#^^a^	*p* = 0.976
	Married	65 (48.5)	28 (53.8)			76 (49.7)	17 (51.5)		
	Divorced	27 (20.1)	10 (19.2)			30 (19.6)	7(21.2)		
	Widowed	24 (17.9)	9 (17.3)			28 (18.3)	5 (15.2)		
**Educational status**	No formal education	23 (17.2)	6 (11.5)	7.140^#^^a^	*p* = 0.068	28 (18.3)	1 (3.0)	13.575 ^#^^a^	*p* = 0.004
	Primary (1-6^th^ grade)	32(23.9)	9 (17.3)			34(22.2)	7 (21.2)		
	Secondary Junior/high school (7-12^th^ grade)	60(44.8)	21 (40.4)			69(45.1)	12 (36.4)		
	College/University (diploma/degree)	19(14.2)	16 (30.8)			22(14.4)	13 (39.4)		
**Family history** ^**b**^	No	110 (82.1)	47 (90.4)	1.379*^a^	*p* = 0.240	126 (82.4)	31 (93.9)	1.959*^a^	*p* = 0.162
	Yes	24 (17.9)	5(9.6)			27 (17.6)	2(6.1)		
**Blood type**	A	45 (33.6)	15 (28.8)	3.955^#^^a^	*p* = 0.138	52 (34.0)	8 (24.2)	1.556^#^^a^	*p* = 0.459
	B & AB^g^	45 (33.6)	12 (23.1)			47 (30.7)	10(30.3)		
	O	44 (32.8)	25 (48.1)			54 (35.3)	15 (45.5)		
**The WHO staging at baseline**	Stage I	18 (13.4)	7 (13.5)	1.867^#^^a^	*p* = 0.601	22 (14.4)	3 (9.1)	1.253^#^^a^	*p* = 0.740
	Stage II	26 (19.4)	12 (23.1)			32 (20.9)	6 (18.2)		
	Stage III	70 (52.2)	22 (42.3)			73 (47.7)	19 (57.6)		
	Stage IV	20 (14.9)	11 (21.2)			26 (17.0)	5 (15.5)		
**TB prophylaxis**	No	117 (87.3)	48 (92.3)	0.51*^a^	*P* = 0.479	134 (87.6)	31 (93.9)	0.553*^a^	*p* = 0.457
	Yes	17 (12.7)	4 (7.7)			19 (12.4)	2 (6.1)		
**ART class regimen**	2 NRTIs +1 INI	68 (50.7)	25 (48.1)	3.925^#^^a^	*P* = 0.141	84 (54.9)	9 (27.3)	8.624^#^^a^	*p* = 0.013
	2 NRTIs + 1 NNRTI	43 (32.1)	23 (44.2)			48 (31.4)	18 (54.5)		
	2NRTIs + 1PI ^d^	23 (17.2)	4 (7.7)			21 (13.7)	6 (18.2)		
**ART regimen as 1**^**st**^**, 2**^**nd**^**, and 3**^**rd**^ **line**	1^st^ line	111 (82.8)	48 (92.3)	1.999*^a^	*P* = 0.157	132 (86.3)	27 (81.8)	0.150*^a^	*p* = 0.699
	2^nd^ and 3^rd^ line^e^	23 (17.2)	4 (7.7)			21 (13.7)	6 (18.2)		
**Change of ART regimen**	No change from the baseline	26 (19.4)	15 (28.8)	3.083^#^^a^	*P* = 0.214	31 (20.3)	10 (30.3)	1.894^#^^a^	*p* = 0.388
	Changed one time	58 (43.3)	16 (30.8)			61 (39.9)	13 (39.4)		
	Changed two or more times^f^	50 (37.3)	21 (40.4)			61 (39.9)	10 (30.3)		
**Current Medications**	ARVs	125 (93.3)	37 (71.2)	14.415*^a^	*p*<0.001	135 (88.2)	27 (81.8)	0.506*^a^	*p* = 0.477
	ARVs with other medications^c^	9 (6.7)	15 (28.8)			18 (11.8)	6 (18.2)		
	Yes	25 (8.7)	55 (24.9)			25 (8.7)	55 (24.9)		
**Coffee-drinking**	No	79 (59.0)	19 (36.5)	6.679*^a^	*P* = 0.006	86 (56.2)	12 (36.4)	3.530*^a^	*p* = 0.060
	Yes	55 (41.0)	33 (63.5)			67 (43.8)	21 (63.6)		
Smoking	No	129 (96.3)	45 (86.5)	4.375^e^^‡^	*P* = 0.039	146 (95.4)	28 (84.8)	2.431^e^^‡^	*p* = 0.041
	Yes^h^	5 (3.7)	7 (13.5)			7 (4.6)	5 (2.7)		

n = 186; Data presented as % prevalence

*Continuity Correction is computed for a 2x2 table

#Pearson Chi-square; ASCVD, atherosclerotic cardiovascular disease; FRS, Framingham risk score; chi-square test); PCE, Pooled Cohort Equation

^‡^ Khat, plant/substance chewed in East-Africa and Middle East as a stimulant or benefit for the purpose of “recreational values”

a. 0 cells (0.0%) had a count of less than 5

b, Family history: History of Cardiometabolic diseases among siblings

c, Other medications include BP lowering drugs, blood sugar lowering agents, lipid lowering agents and antiepileptic and antipsychotics

d, Only one case was on 2 NRTIs + 1INTI +1 PI regimen

e, Only one case was on 3^rd^ line

f, Eleven individual changed 3 times and four participants changed 4 times

g, Fourteen of the cases were with AB blood type

h, 1 cell had a count of less than 5%

‡, Fisher’s Exact Test was used

Logistic regression was utilized to determine predictors of FRS score in both total patients (Table 3) as well as those 40 to 79 years of age (Table 4). When the total patients were considered, younger age (adjusted odds ratio (AOR) 0.20, 95% CI (0.004, 0.091); p< 0.001), lower systolic blood pressure (AOR 0.221 (0.074, 0.605); p< 0.004), and lower total cholesterol (AOR 0.270, 95% CI (0.073, 0.997; p<0.049) were found to be independent predictors of reduced risk for ASCVD (Table 3). Similarly, only younger age (age 40 to 59), the female gender, and lower systolic blood pressure were associated with lower risk for ASCVD among PLWHA age 40 to 79 years using both the FRS (Table 4) and PCE (Table 5).

**Table 3:** Association between ASCVD risk using FRS and clinical characteristics among people living with HIV/AIDS at Zewditu Memorial Hospital, Addis Ababa, Ethiopia.

Clinical characteristics	ASCVD risk using FRS		COR (95% C.I.)	P value	AOR (95% C.I.)	P value
	Low risk (<10%) n = 255	High risk (>10%) n = 33				
**Age (year)**						
≥50	57 (22.4)	31 (93.9)	1.00		1.00	
< 50	198 (77.6)	2 (6.1)	.019 (.004-.080)	*P* < .001	.020 (.004, .091)	*p*<0.001
**CD4 count (cells/μL)**						
>/ = 500	94 (36.9)	13 (39.4)	1.00		1.00	
<500	161 (63.1)	20 (60.6)	.898(.427, 1.889)	*P* = 0.777	.895 (.309, 2.591)	*p* = 0.838
**Systolic blood pressure (mmHg)**						
>/ = 130	89 (34.9)	25 (75.5)	1.00		1.00	
<130	166 (65.1)	8 (24.5)	.172 (.074, .396)	*P*<0.001	.221 (.074, .605)	*p = 0*.004
**Total cholesterol (mg/dL)**						
>/ = 160	164 (64.3)	29 (87.9)	1.00		1.00	
<160	91 (35.7)	4 (12.1)	.249 (.085, 0729)	*P* = 0.011	.270 (.073, .997)	*p = 0*.049
**HDL cholesterol (mg/dL)**						
<50	173 (67.8)	27 81.8)	1.00		1.00	
>/ = 50	82 (32.2)	6 (18.2)	.469 (.186, 1.180)	*P* = 0.*108*	.365 (.106, 1.255)	*p* = 0.110
**Duration on ART (year)**						
>/ = 2 years	229 (89.8)	32 (97)	1.00		1.00	
< 2 years	26 (10.2)	1 (3)	.275 (0.036, 2.098)	*P* = 0.213	.276 (.028, 2.739)	*p* = 0.271
**Blood group**						
Type ‘O’	82 (32.2)	15 (5.2)	1.00		1.00	
Type ‘A’	95 (37.3)	8 (24.2)	.460 (.186, 1.141)	*P* = 0.094	.635 (.193, 2.083	*p* = 0.454
Type ‘B’ & ‘AB’	78 (30.6)	(30.3)10	.701 (.297, 1.653)	*P* = 0.417	1.371 (.418, 4.497)	*p* = 0.603
**Current ARV drug regimens**						
2NRTIs + 1PI*	48 (18.8)	6 (18.2)	1.00		1.00	
2 NRTIs +1 INI	129 (50.6)	9 (27.3)	.323 (.095, 1.097)	*P* = 0.323	.279 (.062, 1.254	*p* = 0.096
2 NRTIs + 1 NNRTI	78 (30.6)	18 54.5)	.535 (.180, 1.593)	*P* = 0.535	1.254 (.304, 5.177)	*p* = 0.754

n = 288; Data presented as % prevalence; ASCVD, Atherosclerotic Cardiovascular Disease; CI, confidence interval; FRS, Framingham risk score; OR, odds ratio

*two of the cases were on 2 NRTIs+ 1INTI +1 PI) regimen.

**Table 4:** Association between ASCVD risk using FRS and clinical characteristics among people living with HIV/AIDS age 40 75 years at Zewditu Memorial Hospital, Addis Ababa, Ethiopia.

Clinical characteristics	ASCVD risk using FRS		COR (95% C.I.)	*P* value	AOR (95% C.I.)	*P* value
	Low risk (<10%)	High risk (≥10%)				
**Number (%)**	153 (82.3)	33 (17.7)				
**Age (year)**						
60–79	14 (9.2)	16 (48.5)	1		1	
40–59	139 (90.8)	17 (51.5)	0.107 (0.045, .257)	*p*<0.001	0.022 (.004, .115)	*p*<0.001
**Gender**						
Male	70 (45.8)	31 (93.9)	1		1	
Female	83 (54.2)	2 (6.1)	0.054 (0.013, .235)	*p*<0.001	0.017 (.002, .126)	*p* < .0001
**CD4 count (cells/μL)**						
>/ = 500	56 (36.6)	13 (39.4)	1		1	
<500	97 (63.4)	20 (60.6)	0.888 (0.410, 1.922)	*P* = 0.763	0.380 (.110, 1.312)	*p* = 0.126
**Systolic blood pressure (mmHg)**						
>/ = 130	71 46.4)	25 (75.8)	1		1	
<130	82 (53.6)	8 (24.2)	0.277 (.118, .653)	*P* = 0.003	0.190 (.054, .668)	*p* = 0.010
**Total cholesterol (mg/dL)**						
>/ = 160	107 (69.9)	29 (87.9)	1		1	
<160	46 (30.1)	4 (12.1)	0.321 (.107, .965)	*P* = 0.043	0.302 (.081, 1.131)	*p* = 0.075
**HDL cholesterol (mg/dL)**						
<40 in male & < 50 in women	63 (41.2)	11 (33.3)	1		1	
>/ = 40 in Male & >/ = 50 in Female	90 (58.8)	22 (66.7)	1.40 (.634, 3.091)	*P* = 0.405	0.308 (.087, 1.095)	*p* = 0.069
**Duration on ART (year)**						
>/ = 2 years	139 (90.8)	32 (18.7)	1		1	
<2 years	14 (9.2)	1 (3.0)	0.310 (.039, 2.446)	*P* = 0.267	0.571 (.046, 7.145)	*p* = 0.664
**Blood group**						
Type ‘O’	54 (35.3)	15 (45.5)	1		1	.
Type ‘A’	52 (34.0)	8 (24.2)	0.554 (.217, 1.416)	*P* = 0.217	0.486 (0.123, 1.924)	*p* = 0.304
Type ‘B’ & ‘AB’	47 (30.7)	10 (30.3)	0.766 (.134, 1.866)	*P* = 0.557	0.824 (0.227, 2.998)	*p* = 0.769
**Current ARV drug regimens**						
2NRTIs + 1PI*	21 (13.7)	6 (18.2)	1	.	1	.
2 NRTIs +1 INI	84 (54.9)	9 (27.3)	0.375 (0.120, 1.171)	*P* = 0.091	0.149 (0.029, .775)	*p* = 0.024
2 NRTIs + 1 NNRTI	48 (31.4)	18 (54.5)	1.313 (0.456, 3.776)	*P* = 0.614	0.450 (.093, 2.177)	*p* = 0.321

n = 186; Data presented as % prevalence; ASCVD, Atherosclerotic Cardiovascular Disease; FRS, Framingham Risk Score; COR, Crude Odds Ratio; AOR, Adjusted Odds Ratio; CI, Confidence Interval

*two of the cases were on 2 NRTIs + 1INTI +1 PI) regimen

**Table 5:** Association between ASCVD risk using PCE and clinical characteristics among people living with HIV/AIDS age 40 to 75 years at Zewditu Memorial Hospital, Addis Ababa, Ethiopia.

Clinical characteristics	ASCVD risk using PCE		COR (95% C.I.)	*P* value	AOR (95% C.I.)	*P* value
	Low risk (<7.5%)	High risk (≥7.5%)				
**Number (%)**	134 (72.0)	52 (28.0)				
**Age (year)**						
60–79	6 (4.5)	24 (46.2)	1		1	
40–59	128 (95.5)	28 (53.8)	0.55 (0.020, 0.146)	*p*<0.001	0.012 (0.002,.066)	*p*<0.001
**Gender**						
Male	62 (46.3)	39 (75.0)	1		1	
Female	72 (53.7)	13 (25.0)	0.287 (0.141, 0.586)	*p* = 0.001	0.185 (0.054,0.630)	*p* = 0.007
**CD4 count (cells/μL)**						
>/ = 500	52 (38.8)	17 (32.7)	1		1	
<500	82 (61.2)	35 (67.3)	1.306 (0.664, 2.566)	*p = 0*.*439*	1.045 (0.385, 2.833)	*p* = 0.931
**Systolic blood pressure (mmHg)**						
>/ = 130	53 (39.6)	43 (82.7)	1		1	
<130	81 (60.4)	9 (17.3)	0.137 (0.062, 0.304)	*p* < .001	0.034 (0.007, 0.162)	*p*<0.001
**Total cholesterol (mg/dL)**						
>/ = 160	99 (73.9)	37 (71.2)	1		1	
<160	35 (26.1)	15 (28.8)	1.147 (0.562,2.340)	*p* = 0.707	1.342 (0.499, 3.607)	*p* = 0.560
**HDL cholesterol (mg/dL)**						
<40 in male & < 50 in women	56 (41.8)	18 (34.6)	1		1	
>/ = 40 in Male & >/ = 50 in Female	78 (58.2)	34 (65.4)	1.356 (0.696, 2.641)	*p* = 0.370	0.635 (0.206, 1.956)	*p* = 0.429
**Duration on ART (year)**						
>/ = 2 years	125 (93.3)	46 (88.5)	1		1	
<2 years	9 (6.7)	6 (11.5)	1.812 (0.611, 5.371)	*p* = 0.284	1.952 (0.365, 10.437)	*p* = 0.434
**Blood group**						
Type ‘O’	44 (32.8)	25 (48.1)	1		1	
Type ‘A’	45 (33.6)	15 (28.8)	0.587 (0.273, 1.258)	*p* = 0.171	0.353 (0.116, 1.073)	*p* = 0.066
Type ‘B’ & ‘AB’	45 (33.6)	12 (23.1)	0.469 (0.210, 1.049)	*p* = 0.065	0.378 (0.122, 1.170)	*p* = 0.092
**Current ARV drug regimens**						
2NRTIs + 1PI*	23 (17.2)	4 (7.7)	1		1	
2 NRTIs +1 INI	68 (50.7)	25 (48.1)	2.114 (0.665, 6.720)	*p* = 0.205	1.600 (0.341, 7.515)	*p* = 0.551
2 NRTIs + 1 NNRTI	43 (32.1)	23 (44.2)	3.076 (0.949, 9.972)	*p* = 0.061	1.820 (0.372, 8.901)	*p* = 0.459

n = 186; Data presented as % prevalence; ASCVD, Atherosclerotic Cardiovascular Disease; FRS, Framingham Risk Score; COR, Crude odds ratio; AOR, adjusted odds ratio; CI, Confidence Interval

*two of the cases were on 2 NRTIs + 1INTI +1 PI) regimen.

**Fig 2:**
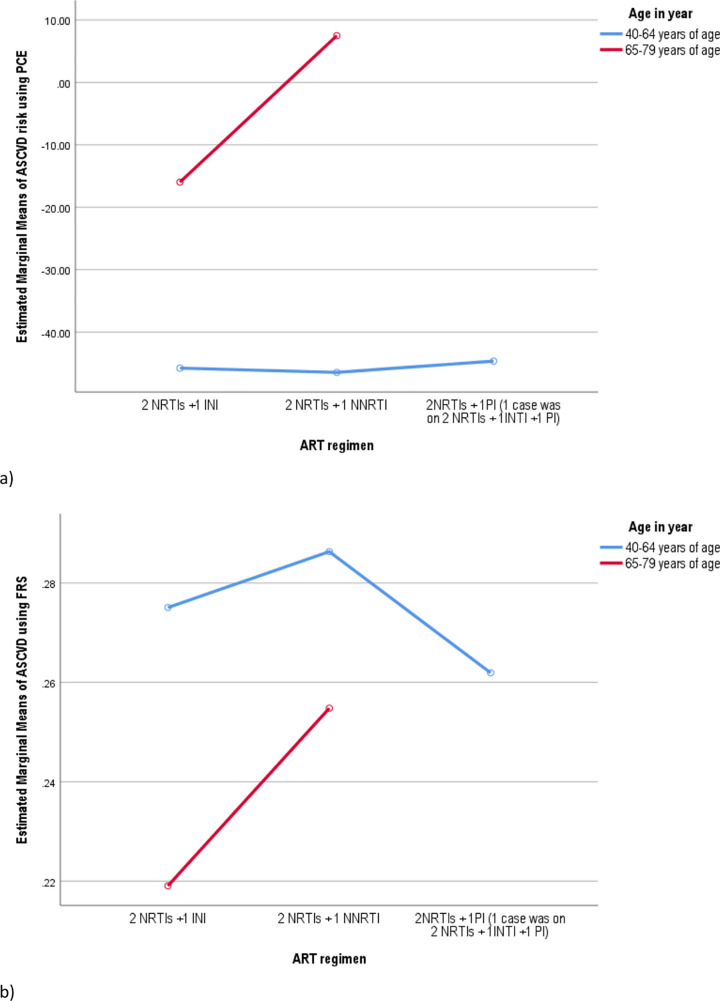
The ASCVD risk based on ART regimen and age interaction among PLWHA age 40–79 years: using the pooled cohort equation (a), and the Framingham risk score (b) estimation.

**Fig 3:**
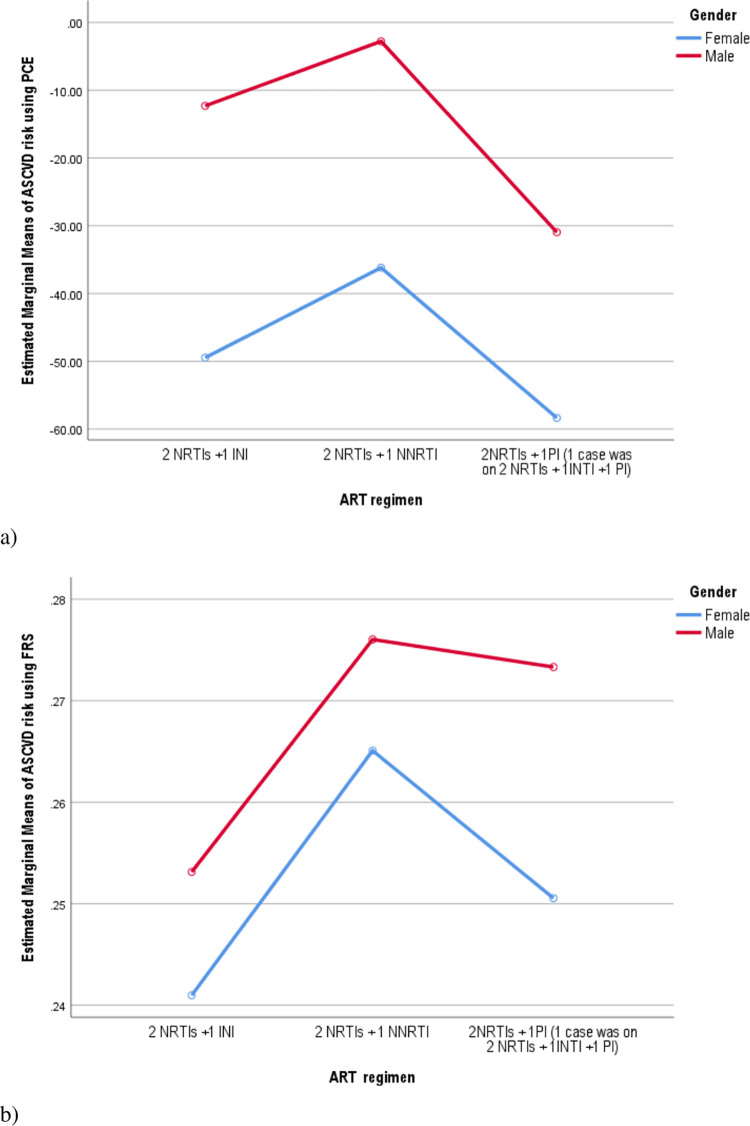
The ASCVD risk based on ART regimen and gender interaction among PLWHA age 40–79 years: using the pooled cohort equation (a), and the Framingham risk score (b) estimation.

**Fig 4:**
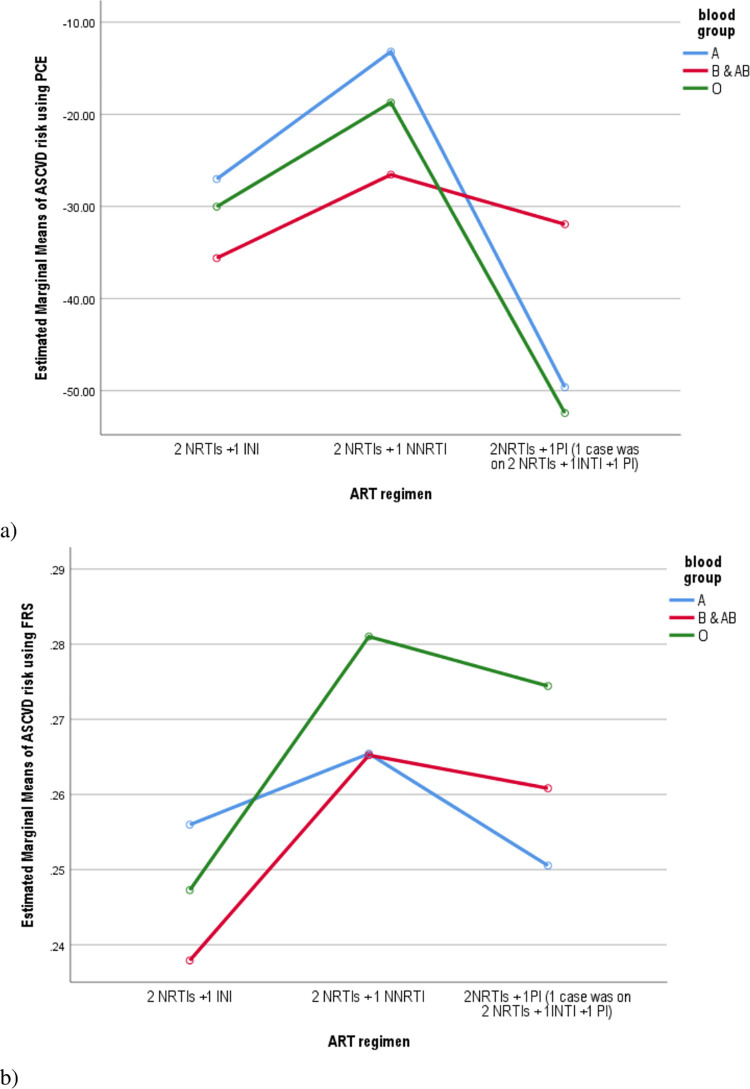
The ASCVD risk based on ART regimen and blood type interaction among PLWHA age 40–79 years: using the pooled cohort equation (a), and the Framingham risk score (b) estimation.

To see the effect of ART regimen on mean score, ASCVD risk was calculated based on FRS and PCE, and a curve was plotted for age (Fig 2), sex (Fig 3), and blood group (Fig 4). The mean score for the risk based on both FRS was found to be higher with NNRTI-based regimen, age 40–64 years, the male gender, and blood type ‘O’. On the other hand, the highest marginal mean was observed in NNRTI based regimen, the male gender, age 65–79 years, and blood type ‘A’ in the PCE method.

## Discussion

This is the first study that attempted to determine the ASCVD risk prevalence and predictors among PLWHA based on both PCE and FRS methods. Several studies investigated the risk of ASCVD in PLWHA using the FRS/PCE tools and reported that both FRS and PCE are equally important in both PLWHA and the general population, although some prefer PCE [3, 17, 18, 32, 33] and others FRS [19, 34]. We used both tools because this is the first study conducted in the country and wanted it to serve as a basis for future similar studies.

The prevalence of elevated risk for ASCVD in all patients based on FRS was 11.5%. It was 28% and 17.7% for those patients 40 to 79 years of age based on the PCE and FRS method, respectively. Since race has been incorporated as a predictor in determination of the risk for ASCVD by the PCE method, an exaggerated prevalence might be seen in such population, as evidenced in the present study (28% for PCE vs. 17.7% for FRS). Moreover, the lower cut-off point used for the PCE method (≥7.5%) could also contribute to the observed higher prevalence.

The prevalence of elevated risk for ASCVD in all patients based on FRS in our study is found to be higher than the Chinese [35], Botswana [36] and Taiwan [37] studies, but lower than the Italian [38] and the US [26] studies. Moreover, the male gender and age ≥50 years were found to be predictors of elevated-risk for ASCVD and this is consistent with studies conducted in the USA [3, 39]. Study design, sample size, population genetics, and study duration could account for the observed discrepancies.

Our study revealed that age plays a major role in the prevalence of elevated ASCVD risk and this is in line with several studies conducted elsewhere [12, 39–41]. In our study, participants with 40 to 64 years of age had a low-risk for ASCVD than those with the age range 65 to 79 years based on both methods and this is in agreement with several studies done globally [10, 12, 23, 37, 42–44]. On the other hand, many other studies have also reported high prevalence of elevated ASCVD risk at 40 to 65 years of age that tended to decline with age above 65 years [44, 45].

Consistent with our finding, there is a sex-based difference in the lifetime risk for ASCVD, being 50% for men and 33.3% for women at 40 years of age and decreasing to 33% for men and 25% for women at 70 years of age [44]. The association of the male gender with increased risk for ASCVD is a subject of controversy. The female sex hormones have been assumed to confer a protective role for ASCVD. However, a recent published study [46] did not provide any evidence for the role of these hormones in ASCVD risk prevention, casting doubt on their protective role. As a result, post-menopausal hormone replacement therapy should not be considered as beneficial for ASCVD prevention strategies. Environmental factors (cigarette smoking and working in hazardous conditions) have also been suggested to play a major role in sex difference of ASCVD distribution [44, 45, 47, 48]. Although females tend to have a lower-risk for ASCVD, its occurrence is associated with poor prognosis and increased risk of mortality [3, 45].

Smoking has been shown to be associated with ASCVD in both PLWHA and the general population [49, 50]. Our study also showed that smoking is a significant predictor of ASCVD in both tools, although the proportion of smokers was less than 5%, which is much lower than other studies conducted elsewhere (23.5%) [51] and 68.7% [49].

Several studies reported that hypertension is an important risk factor for cardiovascular, stroke, and cerbrovascular diseases [52–54]. Mostly hypertension and ASCVD appear together in clinical investigation, although which causes which is a paradox [55]. Lower systolic blood pressure (<130mmHg) in HIV patients was associated with a decreased risk of ASCVD based on FRS and this is comparable with several other studies [56–58]. Systolic blood pressure was also a significant predictor of ASCVD among patients 40 to 79 years of age using both PCE and FRS, suggesting that lowering this pressure to below 130 mmHg could reduce the risk of acquiring ASCVD by 15 to 25%, which is concordant with several studies [16, 59, 60].

Dyslipidemia is considered to be the most frequent risk factor for ASCVD as reported in the literature [58, 61]. A decrease in total cholesterol below 160 mg/dL was associated with a lower risk of ASCVD in all patients in the present study. However, it was not a significant predictor of ASCVD among population age 40 years and above, as reported elsewhere [62].

A significant lower-risk of ASCVD was observed among participants on ART regimen of ‘2 NRTIs +1 INI’ and aged 40 to 79 years using FRS but the PCE-based calculation did not produce any significant association. The lack of association in PCE but not in FRS could be attributed to the difference in the number of lipid variables used by the two methods. FRS uses three different lipids (TC, HDL, and LDL) to predict the risk of ASCVD and cART increase the risk of ASCVD by altering lipid profiles [24, 63].

Between subject analysis for the variables cART, age, gender, and blood group against the risk of ASCVD was performed and the analysis revealed that mean score was highest with NNRTI-based regimen. The association of NNRTIs-based cART with the risk of ASCVD has been widely reported by several studies [64–66], but association was more commonly reported with PI- based cART [66–68]. However, we found no association with PI-based cART, which could possibly be due to the fact that many of the participants were on NNRTIs-based cART until the recent introduction of the integrase inhibitors and/or the small sample size employed.

Only few studies are available that attempted to determine the risk for ASCVD in HIV-infected adults in Africa. Mosepele et al. [36] reported PCE to classify more participants in the elevated risk category than FRS (14.1 vs. 2.6%) and to a similar extent to those having established subclinical atherosclerosis. Mubiru et al [69] conducted a systematic review to find the most frequently used tool for screening the risk and did not find any detectable differences between lipid and non-lipid as well as HIV-specific and non-HIV-specific factors, although they suggested that the inclusion of HIV and ART history might improve accuracy of risk determination. Similarly, a study done in Tanzania by Kingery et al [70] reported that the lifetime and 10-year ASCVD risk as well as the prevalence of metabolic syndrome was higher in ART-experienced than HIV-negative and ART-naïve subjects. All reports highlighted the need for further studies to better understand the risk of CVD in HIV patients, as the burden of the disease is greater in sub-Saharan Africa.

## Limitations of the study

The study may not be used as a representative for the entire PLWHA in Ethiopia as the data were obtained only from a single hospital. The ASCVD risk assessed in this study might not represent an actual ASCVD, as clinical events, or surrogate proofs such as coronary plaque were not determined using computed tomography. The anticipated risk of ASCVD can also be reverted by proper implementation of preventive clinical guidelines during the follow-up period and a healthier life style modification.

## Conclusions

This study highlighted that a significant number of PLWHA are at risk for developing ASCVD in the coming 10 years. In both FRS and PCE, ASCVD was significantly associated with advanced age, male gender, low blood pressure, and smoking. ASCVD management strategies should also take into consideration age, gender, smoking status, and blood pressure control. The ASVD risk calculators, PCE and FRS, have similar prediction capacity in PLWHA. However, PCE might yield an exaggerated prevalence of ASCVD due to the race variable and the lower cut-off point for risk stratification incorporated in the tool. Hence, the tools can be used interchangeably or together.
