# Correction: Predicting the risk of atherosclerotic cardiovascular disease among adults living with HIV/AIDS in Addis Ababa, Ethiopia: A hospital-based study

**DOI:** 10.1371/journal.pone.0326798

**Published:** 2025-06-23

**Authors:** The PLOS One Editors

## Notice of Republication

This article [1] was republished on May 14, 2025, to address an issue identified post-publication. An updated version of S1 File is provided with this notice. Please download this article again to view the correct version.

## Supporting information

S1 File (SAV)
