# Supplementary material for: Predicting the risk of atherosclerotic cardiovascular disease among adults living with HIV/AIDS in Addis Ababa, Ethiopia: A hospital-based study
Source: PLoS One. 2021 Nov 29;16(11):e0260109. doi: 10.1371/journal.pone.0260109 (PMC8629213; doi:10.1371/journal.pone.0260109)
Supplement: Attachment — Submitted filename: Review PLOS ONE 21 18860.docx [file pone.0260109.s002.docx]

**Review	21- 18860 Plos One**

This is a study which assess the prevalence of atherosclerotic cardiovascular disease (ASCVD) risk among a cohort of people living with HIV (PLWHA) in Addis Ababa, Ethiopia. The whole sample includes 288 patients for data analysis. For risk calculation, the authors use two calculators: the Pooled Cohort Equation (PCE) or the Framingham Risk Score (FRS). The results of risk calculation are then reported using both, independently, as well as the analyses of the effect of predictors.

General comments:

The authors should be very careful in all their text that ASCVD risk is assessed in their study, but not ASCVD itself. In more than one place, there is not enough precision on this issue.

I think there could be less acronyms. Please use acronyms if they are frequently used in the text, and try to minimize their use.

A comparison of the risk calculation using both scores would be very helpful, using clinically used values such as low, moderate or elevated risk (or low versus elevated risk). See methodology of : Hernando Knobel 1, Carlos Jericó, Milagro Montero, María L Sorli, Manuela Velat, Ana Guelar, Pere Saballs, Juan Pedro-Botet Global cardiovascular risk in patients with HIV infection: concordance and differences in estimates according to three risk equations (Framingham, SCORE, and PROCAM) AIDS Patient Care STDS . 2007 Jul;21(7):452-7. doi: 10.1089/apc.2006.0165.

Abstract :

Define AOR

Introduction

p 9 line 58. “It is therefore prudent to determine the risk of such CVDs for

59 proper monitoring as well as improving outcomes of cART [13].” Suggest to add “… adequately determine …”

p10 line 73: “The prevalence of ASCVD using FRS reported to be in the range of 70-90% for the low risk, 20-30% for the moderate risk, and 0-20% for the high-risk [17-20].” Is it the inverse of that ? so the authors want to describe the prevalence of cardiovascular risk, or the prevalence of cardiovascular disease ? please be specific, since ASCVD is defined here as atherosclerotic cardiovascular disease (and not atherosclerotic cardiovascular disease risk)

Methods:

Enrolment: described

What were the inclusion and exclusion for enrolment: described

Please in the method, underline that HIV infection was among the inclusion criteria.

How HIV infection was proven ?

p 13, line 132: “Determination of ASCVD among population aged 20 to 132 79 years (n=288) was carried out using the FRS tool.” Please add the work “risk”. The sentence should be “Determination of ASCVD risk among population aged 20 to 132 79 years (n=288) was carried out using the FRS tool.”

Results:

In the Introduction, the authors mention that “The risk can be classified can be classified as low-risk (<10%), moderate risk (10-20%), and 72 high-risk (>20%) using FRS [15], and as low risk (<7.5%) and elevated risk (> 7.5%) using PCE 73 [16].”. However, in Table 1, the risk with FRS seems to be classified as low versus elevated. Please clarify. Please define what is low and what is elevated using FRS.

Same issue: in the introduction it is written as “ .. low risk (<7.5%) and elevated risk (> 7.5%) using PCE ..”. Please, if low and elevated categories are also used for FRS, “FRS” has to be included in the sentence.

p15 line 169 “The calculated risk using both tools presented in Table 2.” Please, specify in this sentence that it is for participants 40 to 79 years.

I do not see Smoking as a predictor in any of the tables. Smoking is a predictor of coronary artery disease (CAD) that is difficult to control in comparative studies assessing causes of CAD in HIV-associated atherosclerosis (see Durand et al. JAIDS. 2011;57(3):245-253, as well as Boldeanu et al. Radiology 2021). Please try to specifically assess the role of smoking in the predicted risk.

Discusssion

3^rd^ paragraph: “The prevalence of the risk for ASCVD in all patients based on FRS was 11.5% and it was 28% and 17.7% for those patients 40 to 79 years of age based on the PCE and FRS method, respectively.” Please add the word elevated: The prevalence of elevated risk for ASCVD in …

Please describe which, if any, studies also describe ASCVD and ASCVD risk in the HIV African populations. Please convince the reader that there a need in the literature for another ASCVD risk assessment study such as this one. Explain what is specific of the present study, in comparison to other published studies.

Please comment and discuss that ASCVD risk is assess in this study, however without available data for proven ASCVD (for example, clinical events, or surrogate proofs such as coronary plaque using computed tomography). This is among the limitations of the study.

References:

ref no 8 seems incomplete

if another reference is needed:

Durand M, Sheehy O, Baril JG, Lelorier J, Tremblay CL. Association between HIV infection, antiretroviral therapy, and risk of acute myocardial infarction: a cohort and nested case-control study using Quebec's public health insurance database. Journal of acquired immune deficiency syndromes (1999). 2011;57(3):245-253.
