# Supplementary material for: Predicting the risk of atherosclerotic cardiovascular disease among adults living with HIV/AIDS in Addis Ababa, Ethiopia: A hospital-based study
Source: PLoS One. 2021 Nov 29;16(11):e0260109. doi: 10.1371/journal.pone.0260109 (PMC8629213; doi:10.1371/journal.pone.0260109)
Supplement: Attachment — Submitted filename: Response to reviewers 2 .docx [file pone.0260109.s004.docx]

**Dear Editors and Reviewers:**

Thank you for your letter and for the reviewers’ comments concerning our manuscript entitled “Predicting the risk of atherosclerotic cardiovascular disease among adults living with HIV/AIDS in Addis Ababa, Ethiopia: a hospital-based study”. The comments are all valuable and very helpful for improving our manuscript. We have seen all the comments carefully and have made correction which we hope meet your expectations. Revised portion are shown with track changes and both the marked and unmarked versions of the manuscript are separately uploaded. The main corrections in the paper and the responses to the Editor’s and reviewer’s comments are as follows:

**Comments of the Editor**

- Review your reference list to ensure that it is complete and correct. Provide rationale for including retracted articles.
- We checked all the references and they are complete and correct. We have included three references (50-52) to that effect. We did not cite any retracted articles.

**Comments of Reviewer 1**

- Removal of the smoking data
- We removed the smoking data because the proportion of smokers was too small. We have now changed the tool from Pearson Chi-Square test to Fisher’s exact test and included the smoking data in the association studies.

Kind regards

Ephrem Engidawork (PhD)

Professor of Pharmacology
