# Supplementary material for: Predicting the risk of atherosclerotic cardiovascular disease among adults living with HIV/AIDS in Addis Ababa, Ethiopia: A hospital-based study
Source: PLoS One. 2021 Nov 29;16(11):e0260109. doi: 10.1371/journal.pone.0260109 (PMC8629213; doi:10.1371/journal.pone.0260109)
Supplement: Peer review history

# Peer review: Decision Letter 0

**DOI:** 10.1371/journal.pone.0260109.r001

**Authors:** Gualtiero I. Colombo

16 Sep 2021

PONE-D-21-18860

Predicting the risk of atherosclerotic cardiovascular disease among adults living with HIV/AIDS in Addis Ababa, Ethiopia: a hospital-based study

PLOS ONE

Dear Dr. Engidawork,

Thank you for submitting your manuscript to PLOS ONE. After careful consideration, we feel that it has merit but does not fully meet PLOS ONE’s publication criteria as it currently stands. Therefore, we invite you to submit a revised version of the manuscript that addresses the points raised during the review process.

The reviewer and this Editor found that the manuscript has merits, but it cannot be accepted in the present form. Specifically: (1) a comparison of the inferred risk derived from the scores used should be included; (2) please, describe enrolment procedures and inclusion and exclusion criteria; (3) please, describe the criteria for HIV infection diagnosis; (4) please, include the smoking habit in the risk model; (5) describe what is the added value/information specific to the present study, in comparison to other published studies; (6) fully describe the limitations of the study.

Please submit your revised manuscript by Oct 31 2021 11:59PM. If you will need more time than this to complete your revisions, please reply to this message or contact the journal office at plosone@plos.org. When you're ready to submit your revision, log on to https://www.editorialmanager.com/pone/ and select the 'Submissions Needing Revision' folder to locate your manuscript file.

Please include the following items when submitting your revised manuscript:

- A rebuttal letter that responds to each point raised by the academic editor and reviewer(s). You should upload this letter as a separate file labeled 'Response to Reviewers'.
- A marked-up copy of your manuscript that highlights changes made to the original version. You should upload this as a separate file labeled 'Revised Manuscript with Track Changes'.
- An unmarked version of your revised paper without tracked changes. You should upload this as a separate file labeled 'Manuscript'.

If you would like to make changes to your financial disclosure, please include your updated statement in your cover letter. Guidelines for resubmitting your figure files are available below the reviewer comments at the end of this letter.

If applicable, we recommend that you deposit your laboratory protocols in protocols.io to enhance the reproducibility of your results. Protocols.io assigns your protocol its own identifier (DOI) so that it can be cited independently in the future. For instructions see: https://journals.plos.org/plosone/s/submission-guidelines#loc-laboratory-protocols. Additionally, PLOS ONE offers an option for publishing peer-reviewed Lab Protocol articles, which describe protocols hosted on protocols.io. Read more information on sharing protocols at https://plos.org/protocols?utm_medium=editorial-email&utm_source=authorletters&utm_campaign=protocols.

We look forward to receiving your revised manuscript.

Kind regards,

Gualtiero I. Colombo, M.D., Ph.D.

Academic Editor

PLOS ONE

Journal requirements:

When submitting your revision, we need you to address these additional requirements.

1. Please ensure that your manuscript meets PLOS ONE's style requirements, including those for file naming. The PLOS ONE style templates can be found at

https://journals.plos.org/plosone/s/file?id=wjVg/PLOSOne_formatting_sample_main_body.pdf and

https://journals.plos.org/plosone/s/file?id=ba62/PLOSOne_formatting_sample_title_authors_affiliations.pdf.

2. Please include additional information regarding the survey or questionnaire used in the study and ensure that you have provided sufficient details that others could replicate the analyses. For instance, if you developed a questionnaire as part of this study and it is not under a copyright more restrictive than CC-BY, please include a copy, in both the original language and English, as Supporting Information.

3. We note that you have stated that you will provide repository information for your data at acceptance. Should your manuscript be accepted for publication, we will hold it until you provide the relevant accession numbers or DOIs necessary to access your data. If you wish to make changes to your Data Availability statement, please describe these changes in your cover letter and we will update your Data Availability statement to reflect the information you provide.

4. Your ethics statement should only appear in the Methods section of your manuscript. If your ethics statement is written in any section besides the Methods, please delete it from any other section.

Additional Editor Comments (if provided):

[Note: HTML markup is below. Please do not edit.]

Reviewers' comments:

Reviewer's Responses to Questions

**Comments to the Author**

1. Is the manuscript technically sound, and do the data support the conclusions?

The manuscript must describe a technically sound piece of scientific research with data that supports the conclusions. Experiments must have been conducted rigorously, with appropriate controls, replication, and sample sizes. The conclusions must be drawn appropriately based on the data presented. 

Reviewer #1: Yes

**********

2. Has the statistical analysis been performed appropriately and rigorously? 

Reviewer #1: Yes

**********

3. Have the authors made all data underlying the findings in their manuscript fully available?

The PLOS Data policy requires authors to make all data underlying the findings described in their manuscript fully available without restriction, with rare exception (please refer to the Data Availability Statement in the manuscript PDF file). The data should be provided as part of the manuscript or its supporting information, or deposited to a public repository. For example, in addition to summary statistics, the data points behind means, medians and variance measures should be available. If there are restrictions on publicly sharing data—e.g. participant privacy or use of data from a third party—those must be specified.

Reviewer #1: Yes

**********

4. Is the manuscript presented in an intelligible fashion and written in standard English?

PLOS ONE does not copyedit accepted manuscripts, so the language in submitted articles must be clear, correct, and unambiguous. Any typographical or grammatical errors should be corrected at revision, so please note any specific errors here.

Reviewer #1: Yes

**********

5. Review Comments to the Author

Please use the space provided to explain your answers to the questions above. You may also include additional comments for the author, including concerns about dual publication, research ethics, or publication ethics. (Please upload your review as an attachment if it exceeds 20,000 characters)

Reviewer #1: Review 21- 18860 Plos One

This is a study which assess the prevalence of atherosclerotic cardiovascular disease (ASCVD) risk among a cohort of people living with HIV (PLWHA) in Addis Ababa, Ethiopia. The whole sample includes 288 patients for data analysis. For risk calculation, the authors use two calculators: the Pooled Cohort Equation (PCE) or the Framingham Risk Score (FRS). The results of risk calculation are then reported using both, independently, as well as the analyses of the effect of predictors.

General comments:

The authors should be very careful in all their text that ASCVD risk is assessed in their study, but not ASCVD itself. In more than one place, there is not enough precision on this issue.

I think there could be less acronyms. Please use acronyms if they are frequently used in the text, and try to minimize their use.

A comparison of the risk calculation using both scores would be very helpful, using clinically used values such as low, moderate or elevated risk (or low versus elevated risk). See methodology of : Hernando Knobel 1, Carlos Jericó, Milagro Montero, María L Sorli, Manuela Velat, Ana Guelar, Pere Saballs, Juan Pedro-Botet Global cardiovascular risk in patients with HIV infection: concordance and differences in estimates according to three risk equations (Framingham, SCORE, and PROCAM) AIDS Patient Care STDS . 2007 Jul;21(7):452-7. doi: 10.1089/apc.2006.0165.

Specific comments in the Word document attached.

**********

6. PLOS authors have the option to publish the peer review history of their article (what does this mean?). If published, this will include your full peer review and any attached files.

If you choose “no”, your identity will remain anonymous but your review may still be made public.

**Do you want your identity to be public for this peer review?** For information about this choice, including consent withdrawal, please see our Privacy Policy.

Reviewer #1: No

[NOTE: If reviewer comments were submitted as an attachment file, they will be attached to this email and accessible via the submission site. Please log into your account, locate the manuscript record, and check for the action link "View Attachments". If this link does not appear, there are no attachment files.]

While revising your submission, please upload your figure files to the Preflight Analysis and Conversion Engine (PACE) digital diagnostic tool, https://pacev2.apexcovantage.com/. PACE helps ensure that figures meet PLOS requirements. To use PACE, you must first register as a user. Registration is free. Then, login and navigate to the UPLOAD tab, where you will find detailed instructions on how to use the tool. If you encounter any issues or have any questions when using PACE, please email PLOS at figures@plos.org. Please note that Supporting Information files do not need this step.

Attachment Submitted filename: Review PLOS ONE 21 18860.docx

# Author comment: Author response to Decision Letter 0

**DOI:** 10.1371/journal.pone.0260109.r002

27 Sep 2021

Dear Editor,

We are very much thankful for the constructive comments and suggestions of the Editor and Reviewers. The comments are all valuable and very helpful for revising and improving our manuscript. We have attended all the comments and provided point-by-point response as depicted below. Changes introduced are marked in red with visible track changes in the manuscript. An unmarked version of the revised paper without track changes has also been submitted separately labeled as “Revised Manuscript without track change'. 

Kind regards,

Ephrem Engidawork (PhD)

Professor of Pharmacology

Editor’s Comment

• Please ensure that your manuscript meets PLOS ONE's style requirements, including those for file naming. 

o We checked and it adheres to the guideline. 

• Please include additional information regarding the survey or questionnaire used in the study and ensure that you have provided sufficient details that others could replicate the analyses. For instance, if you developed a questionnaire as part of this study and it is not under a copyright more restrictive than CC-BY, please include a copy, in both the original language and English, as Supporting Information.

o We used an instrument developed by WHO and can be accesses, as we have given the reference. We have provided sufficient detail about the instrument in the text. 

• We note that you have stated that you will provide repository information for your data at acceptance. Should your manuscript be accepted for publication, we will hold it until you provide the relevant accession numbers or DOIs necessary to access your data. If you wish to make changes to your Data Availability statement, please describe these changes in your cover letter and we will update your Data Availability statement to reflect the information you provide.

o We will submit the requested item after acceptance of the manuscript. 

• Your ethics statement should only appear in the Methods section of your manuscript. If your ethics statement is written in any section besides the Methods, please delete it from any other section.

o It now appears in the Method section. 

Review 21- 18860 Plos One

General comments:

• The authors should be very careful in all their text that ASCVD risk is assessed in their study, but not ASCVD itself. In more than one place, there is not enough precision on this issue. 

o Thank you. Corrected. 

• I think there could be less acronyms. Please use acronyms if they are frequently used in the text, and try to minimize their use. 

o Minimized as much as possible. 

• A comparison of the risk calculation using both scores would be very helpful, using clinically used values such as low, moderate or elevated risk (or low versus elevated risk). See methodology of: Hernando Knobel 1, Carlos Jericó, Milagro Montero, María L Sorli, Manuela Velat, Ana Guelar, Pere Saballs, Juan Pedro-Botet Global cardiovascular risk in patients with HIV infection: concordance and differences in estimates according to three risk equations (Framingham, SCORE, and PROCAM) AIDS Patient Care STDS . 2007 Jul;21(7):452-7. doi: 10.1089/apc.2006.0165.

• Abstract: 

Define AOR

o Explained or defined. 

• Introduction

p 9 line 58. “It is therefore prudent to determine the risk of such CVDs for

59 proper monitoring as well as improving outcomes of cART [13].” Suggest to add “… adequately determine …”

o Corrected accordingly.

p10 line 73: “The prevalence of ASCVD using FRS reported to be in the range of 70-90% for the low risk, 20-30% for the moderate risk, and 0-20% for the high-risk [17-20].” Is it the inverse of that? so the authors want to describe the prevalence of cardiovascular risk, or the prevalence of cardiovascular disease? please be specific, since ASCVD is defined here as atherosclerotic cardiovascular disease (and not atherosclerotic cardiovascular disease risk)

o The percentages are correct. The most prevalent form of ASCVD risk is ‘Low risk’. Corrections are also made as per the comment

• Methods:

Enrolment: 

o described

What were the inclusion and exclusion for enrolment? 

o described

Please in the method, underline that HIV infection was among the inclusion criteria.

o Accommodated

How HIV infection was proven?

o Confirmation of HIV was already made based on laboratory investigation as documented in patients’ charts. 

p 13, line 132: “Determination of ASCVD among population aged 20 to 132 79 years (n=288) was carried out using the FRS tool.” Please add the work “risk”. The sentence should be “Determination of ASCVD risk among population aged 20 to 132 79 years (n=288) was carried out using the FRS tool.” 

o Accommodated.

• Results:

In the Introduction, the authors mention that “The risk can be classified as low-risk (<10%), moderate risk (10-20%), and high-risk (>20%) using FRS [15], and as low risk (<7.5%) and elevated risk (> 7.5%) using PCE 73 [16].”. However, in Table 1, the risk with FRS seems to be classified as low versus elevated. Please clarify. Please define what is low and what is elevated using FRS. 

o Corrected.

Same issue: in the introduction it is written as “ .. low risk (<7.5%) and elevated risk (> 7.5%) using PCE.”. Please, if low and elevated categories are also used for FRS, “FRS” has to be included in the sentence.

o Corrected.

p15 line 169 “The calculated risk using both tools presented in Table 2.” Please, specify in this sentence that it is for participants 40 to 79 years.

o It is already provided in the footnote. 

I do not see Smoking as a predictor in any of the tables. Smoking is a predictor of coronary artery disease (CAD) that is difficult to control in comparative studies assessing causes of CAD in HIV-associated atherosclerosis (see Durand et al. JAIDS. 2011;57(3):245-253, as well as Boldeanu et al. Radiology 2021). Please try to specifically assess the role of smoking in the predicted risk. 

o We opted to remove the smoking data from our results because we found the expected count was less than 5%, which is because of the smaller number of smokers in the cohort

• Discussion

3rd paragraph: “The prevalence of the risk for ASCVD in all patients based on FRS was 11.5% and it was 28% and 17.7% for those patients 40 to 79 years of age based on the PCE and FRS method, respectively.” Please add the word elevated: The prevalence of elevated risk for ASCVD in …

o Amended.

Please describe which, if any, studies also describe ASCVD and ASCVD risk in the HIV African populations. Please convince the reader that there a need in the literature for another ASCVD risk assessment study such as this one. Explain what is specific of the present study, in comparison to other published studies. 

o Revised as per the comment.

Please comment and discuss that ASCVD risk is assessed in this study, however without available data for proven ASCVD (for example, clinical events, or surrogate proofs such as coronary plaque using computed tomography). This is among the limitations of the study.

o Accommodated.

• References:

ref no 8 seems incomplete

if another reference is needed:

Durand M, Sheehy O, Baril JG, Lelorier J, Tremblay CL. Association between HIV infection, antiretroviral therapy, and risk of acute myocardial infarction: a cohort and nested case-control study using Quebec's public health insurance database. Journal of acquired immune deficiency syndromes (1999). 2011;57(3):245-253.

o The Reference. Authors name was modified.

Attachment Submitted filename: Response to reviewers.docx

# Peer review: Decision Letter 1

**DOI:** 10.1371/journal.pone.0260109.r003

**Authors:** Gualtiero I. Colombo

11 Oct 2021

PONE-D-21-18860R1Predicting the risk of atherosclerotic cardiovascular disease among adults living with HIV/AIDS in Addis Ababa, Ethiopia: a hospital-based studyPLOS ONE

Dear Dr. Engidawork,

Thank you for submitting your manuscript to PLOS ONE. After careful consideration, we feel that it has merit but does not fully meet PLOS ONE’s publication criteria as it currently stands. Therefore, we invite you to submit a revised version of the manuscript that addresses the points raised during the review process.

I recommend that the authors discuss how the absence of data on smoking habits may affect their results.

Please submit your revised manuscript by Nov 25 2021 11:59PM. If you will need more time than this to complete your revisions, please reply to this message or contact the journal office at plosone@plos.org. When you're ready to submit your revision, log on to https://www.editorialmanager.com/pone/ and select the 'Submissions Needing Revision' folder to locate your manuscript file.

Please include the following items when submitting your revised manuscript:

- A rebuttal letter that responds to each point raised by the academic editor and reviewer(s). You should upload this letter as a separate file labeled 'Response to Reviewers'.
- A marked-up copy of your manuscript that highlights changes made to the original version. You should upload this as a separate file labeled 'Revised Manuscript with Track Changes'.
- An unmarked version of your revised paper without tracked changes. You should upload this as a separate file labeled 'Manuscript'.

If you would like to make changes to your financial disclosure, please include your updated statement in your cover letter. Guidelines for resubmitting your figure files are available below the reviewer comments at the end of this letter.

If applicable, we recommend that you deposit your laboratory protocols in protocols.io to enhance the reproducibility of your results. Protocols.io assigns your protocol its own identifier (DOI) so that it can be cited independently in the future. For instructions see: https://journals.plos.org/plosone/s/submission-guidelines#loc-laboratory-protocols. Additionally, PLOS ONE offers an option for publishing peer-reviewed Lab Protocol articles, which describe protocols hosted on protocols.io. Read more information on sharing protocols at https://plos.org/protocols?utm_medium=editorial-email&utm_source=authorletters&utm_campaign=protocols.

We look forward to receiving your revised manuscript.

Kind regards,

Gualtiero I. Colombo, M.D., Ph.D.

Academic Editor

PLOS ONE

Journal Requirements:

Please review your reference list to ensure that it is complete and correct. If you have cited papers that have been retracted, please include the rationale for doing so in the manuscript text, or remove these references and replace them with relevant current references. Any changes to the reference list should be mentioned in the rebuttal letter that accompanies your revised manuscript. If you need to cite a retracted article, indicate the article’s retracted status in the References list and also include a citation and full reference for the retraction notice.

Additional Editor Comments (if provided):

[Note: HTML markup is below. Please do not edit.]

Reviewers' comments:

Reviewer's Responses to Questions

**Comments to the Author**

1. If the authors have adequately addressed your comments raised in a previous round of review and you feel that this manuscript is now acceptable for publication, you may indicate that here to bypass the “Comments to the Author” section, enter your conflict of interest statement in the “Confidential to Editor” section, and submit your "Accept" recommendation.

Reviewer #1: (No Response)

**********

2. Is the manuscript technically sound, and do the data support the conclusions?

The manuscript must describe a technically sound piece of scientific research with data that supports the conclusions. Experiments must have been conducted rigorously, with appropriate controls, replication, and sample sizes. The conclusions must be drawn appropriately based on the data presented. 

Reviewer #1: Yes

**********

3. Has the statistical analysis been performed appropriately and rigorously? 

Reviewer #1: Yes

**********

4. Have the authors made all data underlying the findings in their manuscript fully available?

The PLOS Data policy requires authors to make all data underlying the findings described in their manuscript fully available without restriction, with rare exception (please refer to the Data Availability Statement in the manuscript PDF file). The data should be provided as part of the manuscript or its supporting information, or deposited to a public repository. For example, in addition to summary statistics, the data points behind means, medians and variance measures should be available. If there are restrictions on publicly sharing data—e.g. participant privacy or use of data from a third party—those must be specified.

Reviewer #1: Yes

**********

5. Is the manuscript presented in an intelligible fashion and written in standard English?

PLOS ONE does not copyedit accepted manuscripts, so the language in submitted articles must be clear, correct, and unambiguous. Any typographical or grammatical errors should be corrected at revision, so please note any specific errors here.

Reviewer #1: Yes

**********

6. Review Comments to the Author

Please use the space provided to explain your answers to the questions above. You may also include additional comments for the author, including concerns about dual publication, research ethics, or publication ethics. (Please upload your review as an attachment if it exceeds 20,000 characters)

Reviewer #1: Comment of reviewer (in submission version):

I do not see Smoking as a predictor in any of the tables. Smoking is a predictor of coronary artery disease (CAD) that is difficult to control in comparative studies assessing causes of CAD in HIV-associated atherosclerosis (see Durand et al. JAIDS. 2011;57(3):245-253, as well as Boldeanu et al. Radiology 2021). Please try to specifically assess the role of smoking in the predicted risk.

Answer of authors:

We opted to remove the smoking data from our results because we found the

expected count was less than 5%, which is because of the smaller number of smokers

in the cohort

New comment in R1 :

Please, the removal of smoking data from the results should be discussed in the Discussion section, as well as how this could affect your results, since this predictor seems to be difficult to control in comparative studies assessing causes of CAD in HIV-associated atherosclerosis, and since it is an important predictor of CAD.

**********

7. PLOS authors have the option to publish the peer review history of their article (what does this mean?). If published, this will include your full peer review and any attached files.

If you choose “no”, your identity will remain anonymous but your review may still be made public.

**Do you want your identity to be public for this peer review?** For information about this choice, including consent withdrawal, please see our Privacy Policy.

Reviewer #1: No

[NOTE: If reviewer comments were submitted as an attachment file, they will be attached to this email and accessible via the submission site. Please log into your account, locate the manuscript record, and check for the action link "View Attachments". If this link does not appear, there are no attachment files.]

While revising your submission, please upload your figure files to the Preflight Analysis and Conversion Engine (PACE) digital diagnostic tool, https://pacev2.apexcovantage.com/. PACE helps ensure that figures meet PLOS requirements. To use PACE, you must first register as a user. Registration is free. Then, login and navigate to the UPLOAD tab, where you will find detailed instructions on how to use the tool. If you encounter any issues or have any questions when using PACE, please email PLOS at figures@plos.org. Please note that Supporting Information files do not need this step.

# Author comment: Author response to Decision Letter 1

**DOI:** 10.1371/journal.pone.0260109.r004

12 Oct 2021

Dear Editors and Reviewers: 

Thank you for your letter and for the reviewers’ comments concerning our manuscript entitled “Predicting the risk of atherosclerotic cardiovascular disease among adults living with HIV/AIDS in Addis Ababa, Ethiopia: a hospital-based study”. The comments are all valuable and very helpful for improving our manuscript. We have seen all the comments carefully and have made correction which we hope meet your expectations. Revised portion are shown with track changes and both the marked and unmarked versions of the manuscript are separately uploaded. The main corrections in the paper and the responses to the Editor’s and reviewer’s comments are as follows:

Comments of the Editor

• Review your reference list to ensure that it is complete and correct. Provide rationale for including retracted articles.

� We checked all the references and they are complete and correct. We have included three references (50-52) to that effect. We did not cite any retracted articles. 

Comments of Reviewer 1

• Removal of the smoking data 

� We removed the smoking data because the proportion of smokers was too small. We have now changed the tool from Pearson Chi-Square test to Fisher’s exact test and included the smoking data in the association studies. 

Kind regards

Ephrem Engidawork (PhD)

Professor of Pharmacology

Attachment Submitted filename: Response to reviewers 2 .docx

# Editor report: Decision Letter 2

**DOI:** 10.1371/journal.pone.0260109.r005

**Authors:** Gualtiero I. Colombo

3 Nov 2021

Predicting the risk of atherosclerotic cardiovascular disease among adults living with HIV/AIDS in Addis Ababa, Ethiopia: a hospital-based study

PONE-D-21-18860R2

Dear Dr. Engidawork,

We’re pleased to inform you that your manuscript has been judged scientifically suitable for publication and will be formally accepted for publication once it meets all outstanding technical requirements.

Within one week, you’ll receive an e-mail detailing the required amendments. When these have been addressed, you’ll receive a formal acceptance letter and your manuscript will be scheduled for publication.

An invoice for payment will follow shortly after the formal acceptance. To ensure an efficient process, please log into Editorial Manager at http://www.editorialmanager.com/pone/, click the 'Update My Information' link at the top of the page, and double check that your user information is up-to-date. If you have any billing related questions, please contact our Author Billing department directly at authorbilling@plos.org.

If your institution or institutions have a press office, please notify them about your upcoming paper to help maximize its impact. If they’ll be preparing press materials, please inform our press team as soon as possible -- no later than 48 hours after receiving the formal acceptance. Your manuscript will remain under strict press embargo until 2 pm Eastern Time on the date of publication. For more information, please contact onepress@plos.org.

Kind regards,

Gualtiero I. Colombo, M.D., Ph.D.

Academic Editor

PLOS ONE

Additional Editor Comments (optional):

Reviewers' comments:

# Editor report: Acceptance letter

**DOI:** 10.1371/journal.pone.0260109.r006

**Authors:** Gualtiero I. Colombo

12 Nov 2021

PONE-D-21-18860R2 

Predicting the risk of atherosclerotic cardiovascular disease among adults living with HIV/AIDS in Addis Ababa, Ethiopia: a hospital-based study  

Dear Dr. Engidawork:

I'm pleased to inform you that your manuscript has been deemed suitable for publication in PLOS ONE. Congratulations! Your manuscript is now with our production department. 

If your institution or institutions have a press office, please let them know about your upcoming paper now to help maximize its impact. If they'll be preparing press materials, please inform our press team within the next 48 hours. Your manuscript will remain under strict press embargo until 2 pm Eastern Time on the date of publication. For more information please contact onepress@plos.org.

If we can help with anything else, please email us at plosone@plos.org. 

Thank you for submitting your work to PLOS ONE and supporting open access. 

Kind regards, 

PLOS ONE Editorial Office Staff

on behalf of

Dr. Gualtiero I. Colombo 

Academic Editor

PLOS ONE
